# Effect of Vortioxetine in Comparison to Fluoxetine on Metabolic Parameters in Patients With Depressive Disorder: A Randomized Controlled Trial

**DOI:** 10.7759/cureus.53178

**Published:** 2024-01-29

**Authors:** Karthik Sankar, Abdul Ajeed Mohathasim Billah, Natrajan Shanmugasundram, Sankar Veintramuthu, Sushma Viswanathan

**Affiliations:** 1 Pharmacy, Sri Ramachandra Institute of Higher Education and Research, Chennai, IND; 2 Psychiatry, Sri Ramachandra Institute of Higher Education and Research, Chennai, IND; 3 Pharmaceutics, PSG College of Pharmacy, Coimbatore, IND

**Keywords:** adverse drug reaction (adr), serotonin modulator, selective serotonin reuptake inhibitor (ssri), major depression disorder, metabolic syndrome (metsy)

## Abstract

Background

Major depressive disorder (MDD) is a debilitating mood disorder that increases the risk of metabolic syndrome (MS), emphasizing the need for mental and physical health treatments. Although many studies have linked atypical antipsychotics to metabolic disturbances, there is limited evidence linking selective serotonin reuptake inhibitor use to MS. This study aimed to assess the risk of MS among patients with MDD who were administered vortioxetine and fluoxetine.

Methodology

This was a prospective, open-label, randomized controlled trial conducted in the psychiatry department. Using computer-generated random numbers, the physician assigned fluoxetine 20 mg or vortioxetine 10 mg and recorded MS parameters at baseline and each visit (4, 8, 12, 16, 20, and 24 weeks). This study was registered with CTRI (CTRI/2021/07/034892).

Results

A total of 122 participants were allocated randomly to the following two groups: group A (n = 60) and group B (n = 62). An independent-sample t-test showed a significant improvement in fasting plasma glucose (FPG) at week eight (p = 0.005), triglycerides (TGs) at week 16 (p = 0.005), high-density lipoprotein (HDL) at week 20 (p = 0.005), and waist circumference at week 24 (p = 0.005) in group A compared to group B. However, systolic blood pressure (SBP) and diastolic blood pressure (DBP) were not significantly associated with either group (p = 0.126 and p = 0.793, respectively). Overall depression remission (Hamilton Depression Rating Scale (HAM-D)) and medication adherence rating scale scores were similar between groups (p = 0.337 and 0.325, respectively). Furthermore, most adverse drug reactions were possibly associated with the study drugs.

Conclusions

In comparison to group B, group A showed significant improvements in FPG, HDL, and waist circumference more effectively; however, both groups led to higher TG levels, with non-significant numerical improvements observed in SBP and DBP in both groups. In addition, both treatment groups reduced the HAM-D score and had a similar MDD remission rate.

## Introduction

Depression is a devastating mental illness characterized by mood disorders, commonly referred to as major depressive disorder (MDD), clinical depression, or melancholia [1]. Depression is a prevalent condition among humans [2]. Based on recent World Health Organization (WHO) data, MDD affects approximately 3.8% of the global population, with an estimated 280 million individuals worldwide affected by this mental health condition [3]. Adherence to antidepressants is crucial for effectively managing MDD. The effectiveness of antidepressants and their influence on quality of life remain uncertain [4,5]. Additionally, the relationship between drug adherence and improvements in depressive symptoms is poorly understood [6]. The risk of developing metabolic syndrome (MS) is increased by twice in people who are suffering from MDD, further underscoring the importance of finding effective treatment options that address both mental and physical health [7]. A few studies have explored the association between these variables with inconsistent findings. Several studies have reported inconclusive findings regarding the association between depression and MS [8,9]; however, one study found a significant correlation between MS parameters and depression [10]. A meta-analysis reported that the risk of MS was 1.5 times greater in individuals who suffer from MDD compared to those without MDD. The global prevalence of MS has risen, and it is estimated to affect about 20-25% of the global population [11]. The prevalence of MS in Indian adults has been estimated at 30% [12]. Antidepressants may have a direct influence on MS, independent of psychiatric disorders [13]. Although numerous studies have established that atypical antipsychotics are associated with a higher risk of MS, there is a paucity of available data to provide evidence linking selective serotonin reuptake inhibitor (SSRI) use and MS [14]. Studies have investigated the metabolic parameters associated with SSRI use and found an increase in obesity and hypercholesterolemia [15]. There exists a body of consistent empirical evidence indicating that the use of antidepressant medications, in particular, serotonin and norepinephrine reuptake inhibitors and tricyclic antidepressants, is linked to a rise in cardiac vagal control. Additionally, it has been observed that antidepressant users may experience an increase in abdominal obesity, which, in turn, contributes to elevated systolic blood pressure (SBP) and diastolic blood pressure (DBP) [16]. In this context, contemporary research suggests that vortioxetine, an antidepressant, functions as a serotonin modulator and has been observed to mitigate adverse effects and enhance cognitive function [17]. The main objective of this investigation was to ascertain the comparative risk of MS between patients with MDD treated with vortioxetine tablets and fluoxetine capsules using the International Diabetes Federation (IDF) criteria.

## Materials and methods

Study design and site

A prospective, open-label, randomized controlled trial (RCT) was conducted in the Department of Psychiatry at the Sri Ramachandra Hospital, Chennai. The participants were allocated into two groups using a computer-generated list of random numbers under the supervision of a medical practitioner from the psychiatry department. The two groups were administered either tablet vortioxetine 10 mg or capsule fluoxetine 20 mg, and the baseline data were recorded and followed up for 24 weeks. The study was performed from February 2022 to January 2023 over a 12-month period.

Ethical considerations

Participation was voluntary, with patients and caregivers being duly informed about the research objectives. The study protocol was approved by the institutional ethics committee (approval number: IEC/20/SEP/158/33). This study was prospectively registered with the Clinical Trial Registry, India (CTRI/2021/07/034892). Participants provided written informed consent, and the investigation was carried out following the Indian Council of Medical Research’s (ICMR) revised National Ethical Guidelines for Biomedical and Health Research Involving Human Participants in 2017.

Sample size

The sample size was determined using nMaster software version 29.0 at a power of 0.80, an alpha error of 0.05, and an effect size of 0.4656, with the standard deviation (SD) of group A being 7.0 and group B being 8.0 with a 95% confidence interval (CI). The required sample size per group was 60. Considering an attrition rate of 15%, about 69 participants were recruited in each group.

Study participants

We included individuals between the ages of 18 and 50 years, of both genders, with a diagnosis of mild-to-moderate MDD, based on the Fifth Edition of the Diagnostic and Statistical Manual of Mental Disorders, and with Hamilton Depression Scale (HAM-D) scores between ≥7 and ≤24. Patients were recruited if they provided both verbal and written informed consent to participate in the study. We excluded those who had a concomitant neurological illness, were currently taking psychoactive medications, had abnormal metabolic risk parameters as per IDF criteria (triglycerides (TGs) levels above ≥150 mg/dL, high-density lipoprotein (HDL) <40 mg/dL in males and <50 mg/dL in females, fasting plasma glucose (FPG) 100 mg/dL, and blood pressure (BP) ≥130/85 mm Hg [18]), were known cases of type 2 diabetes, obesity, valvular disease, sleep disorder, polycystic ovarian disease, substance abuse, alcohol abuse, eating disorders, dieting, known allergies with study drugs, and pregnant and lactating women. Furthermore, patients who had undergone electroconvulsive therapy within the past six months and those exhibiting active suicidal thoughts were also excluded.

Clinical outcome measures

The participants in this study completed a standardized data collection form to gather information on demographic characteristics (such as gender, age, education level, occupation, and marital status) and clinical data regarding study drugs, personal habits, diabetes-related complications, and concurrent medical comorbidities. MDD was assessed using the HAM-D questionnaire. We obtained various anthropometric factors, such as weight, waist size, SBP, and DBP. Furthermore, 10 mL of whole blood was collected from patients by a phlebotomist to assess the following biochemical parameters: FPG, HDL, and TGs. Clinical and biochemical assessments were conducted at two time points, namely, before starting antidepressant therapy and then at four-week intervals until week 24.

Study procedure

Patients who agreed to provide informed consent and met the inclusion criteria were assessed by a psychiatrist. Subsequently, the study participants were randomly assigned to receive either tablet vortioxetine (10 mg/day) or capsule fluoxetine (20 mg/day). All participants were instructed to notify the researchers regarding any alterations in their dietary habits or appetite and were monitored for 24 weeks. The baseline data of waist circumference, FPG, BP, HDL, TGs, and HAM-D score were measured before the start of the drug treatment as well as week 4, 8, 12, 16, 20, and 24 after the start of either fluoxetine 20 mg or vortioxetine 10 mg. The reference ranges were followed according to IDF criteria. Similar to the MS parameters, the medication adherence of the patients was assessed using the Medication Adherence Report Scale (MARS) questionnaire, with scores ranging from 0 to 10, with higher scores indicating better adherence. Similarly, the causality of adverse drug reactions (ADRs) was also assessed using the WHO ADR probability scale during the study period.

Statistical analysis

The data were meticulously analyzed using SPSS Statistics for Windows, version 29.0 (IBM Corp., Armonk, NY, USA). Descriptive statistics were employed to analyze the findings, applying frequency and percentage for categorical variables and mean and standard deviation for continuous variables. The independent-sample t-test was used to determine significant differences between bivariate samples. The study applied repeated-measures analysis of variance (ANOVA) with Bonferroni correction to control type I errors across multiple comparisons. The chi-square test assessed the statistical significance of categorical data. All statistical tests were two-tailed, with the significance level set at p-values <0.05.

## Results

**Figure 1:**
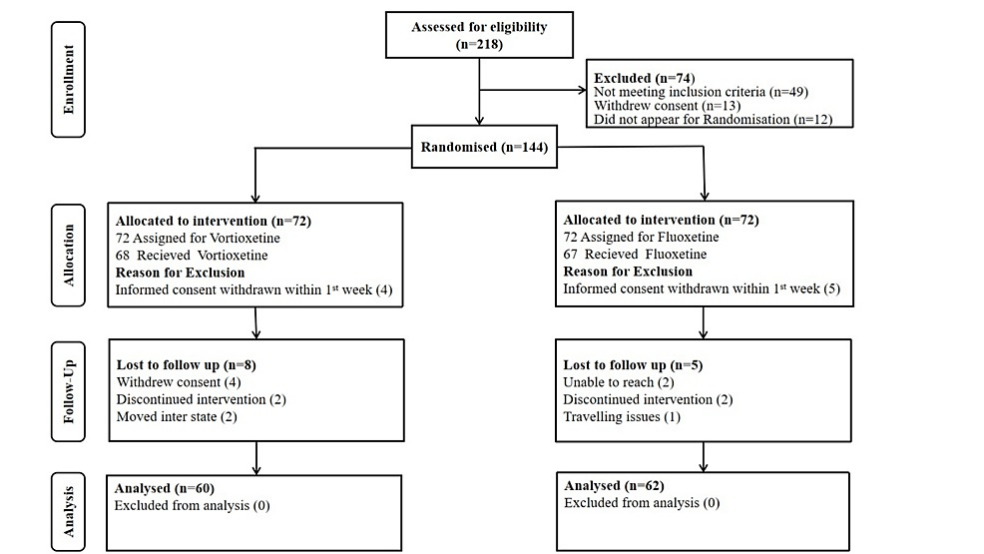
CONSORT flow diagram. CONSORT: Consolidated Standards of Reporting Trials

A total of 218 patients were subjected to a comprehensive screening in this study. About 74 participants were excluded from the study as they failed to fulfill the inclusion criteria (n = 49), withdrew their consent (n = 13), and did not show a willingness to participate in randomization (n = 12). The remaining 144 patients were randomly assigned to one of the two treatment groups. Of them, 22 dropped out for various reasons, including withdrawal of consent (n = 13), being unable to reach the patients after the first week of the trial (n = 2), travel-related problems (n = 1), discontinued interventions (n = 4), and interstate relocation (n = 2). Ultimately, we analyzed 122 patients, with 60 in group A and 62 in group B (Figure 1). The demographic and clinical characteristics of the participants are presented in (Table 1). Throughout the study, there were no discernible changes in the patients’ appetite or food intake. This conclusion was reached after examining their responses to a standardized questionnaire that assessed their intake of carbohydrates, lipids, and proteins as well as their appetite at each visit.

**Table 1:** Baseline characteristics of the study population. Continuous variables are expressed as the mean ± standard deviation. The categorical values are presented as n (%). Group A: vortioxetine 10 mg. Group B: fluoxetine 20 mg HAM-D: Hamilton Depression Rating Scale; FPG: fasting plasma glucose; TGs: triglycerides; HDL: high-density lipoprotein; SBP: systolic blood pressure; DBP: diastolic blood pressure

Characteristics	Group A (N = 60)	Group B (N = 62)	P-value
Age (year)	36.98 ± 6.9	38.86 ± 8.6	0.112
Gender			
Male	29 (48.3%)	32 (51.61%)	0.621
Female	31 (51.7%)	30 (48.39%)	
Occupation			
Employed	28 (46.7%)	27 (43.5%)	0.454
Unemployed	11 (18.3%)	15 (24.2%)	
Business	9 (15.0%)	5 (8.1%)	
Daily wages	10 (16.7%)	12 (19.4%)	
Pensioner	2 (3.3%)	3 (4.8%)	
Education			
Middle/High school	16 (26.7%)	14 (22.6%)	0.368
Undergraduate	21 (35.0%)	22 (35.5%)	
Postgraduate	23 (38.3%)	26 (41.9%)	
Marital status			
Single	25 (41.7%)	23 (37.1%)	0.232
Married	20 (33.3%)	22 (35.5%)	
Separated	6 (10.0%)	8 (12.9%)	
Divorced	4 (6.7%)	6 (9.7%)	
Widowed	5 (8.3%)	3 (4.8%)	
Family type			
Nuclear	24 (40.0%)	25 (40.3%)	0.542
Joint	25 (41.7%)	24 (38.7%)	
Living alone	11 (18.3%)	13 (21.0%)	
Socioeconomic class			
Upper	7 (11.7%)	4 (6.5%)	0.381
Upper middle	12 (20.0%)	8 (12.9%)	
Lower middle	29 (48.3%)	27 (43.5%)	
Upper lower	8 (13.3%)	16 (25.8%)	
Lower	4 (6.7%)	7 (11.3%)	
Duration of depression (year)	2.3 (1.0)	2.4 (1.1%)	0.145
HAM-D score	18.4 ± 3.6	17.9 ± 3.6	0.213
FPG (mg/dL)	93.3 ± 6.9	94.9 ± 6.1	0.322
TGs (mg/dL)	137.8 ± 6.9	137.2 ± 7.6	0.517
HDL (mg/dL)	53.1 ± 8.1	53.7 ± 7.2	0.662
Blood pressure			
SBP (mmHg)	125.1 ± 4.2	124.6 ± 5.4	0.785
DBP (mmHg)	83.1 ± 2.1	82.4 ± 2.8	0.124

The risk parameters for MS, such as waist circumference, FPG, TGs, HDL, and BP (SBP and DBP), showed similar biochemical and anthropometric characteristics in both groups at baseline during the study period. A comparison was made using an independent-sample t-test between the two groups concerning the impact of the study drugs. The mean ± SD values indicated a significant association in FPG at week eight (p = 0.005), TGs at week 16 (p = 0.005), HDL at week 20 (p = 0.005), and waist circumference at week 24 (p = 0.005). However, no meaningful association was found between the study drugs and SBP (p = 0.126) or DBP (p = 0.793). The results of the independent-sample t-test are shown in (Table 2). Within-group analysis using repeated-measures ANOVA test was performed and compared the mean scores from baseline followed by weeks 4, 8, 12, 16, 20, and 24. The study found that in group A, there was a significant decrease in FPG (97.30 ± 10.98 to 90.80 ± 9.82), HDL (53.43 ± 8.53 to 56.10 ± 7.51), and waist circumference (83.06 ± 5.66 to 81.45 ± 5.72), while there was a significant (p < 0.05) increase in mean TG level (137.26 ± 7.69 to 154.25 ± 11.89). Similarly, the mean FPG and HDL in group B decreased significantly from 98.98 ± 6.24 to 93.97 ± 10.32 and from 53.77 ± 7.31 to 51.44 ± 7.13, respectively (p < 0.05). However, there was a significant increase in the mean scores for TGs (138.83 ± 6.98 to 157.40 ± 10.09) and waist circumference (81.27 ± 7.17 to 84.75 ± 7.27) (p < 0.05). Furthermore, a statistically insignificant difference (p > 0.05) was observed with SBP and DBP, with the only numerical improvement being greater in group A than in group B.

**Table 2:** Between-group comparison of metabolic parameters in patients with major depressive disorder. Values are presented as mean ± standard deviation. Group A: vortioxetine 10 mg. Group B: fluoxetine 20 mg. The between-group comparisons at each visit were made with the independent-sample t-test. WC: waist circumference; FPG: fasting plasma glucose; TGs: triglycerides; HDL: high-density lipoprotein; SBP: systolic blood pressure; DBP: diastolic blood pressure

Metabolic parameters	Treatment period (week)	Group A	Group B	P-value
WC (cm)	0	83.06 ± 5.6	81.27 ± 7.1	0.131
	24	81.45 ± 5.7	84.75 ± 7.2	0.005
FPG (mg/dL)	0	97.30 ± 10.9	98.98 ± 6.2	0.163
	24	90.80 ± 9.8	93.9 ± 10.3	0.003
TGs (mg/dL)	0	137.26 ±7 .6	138.8 ± 6.9	0.667
	24	154.25 ±11.8	157.4 ± 10.0	0.001
HDL (mg/dL)	0	53.43 ± 8.5	53.77 ± 7.3	0.813
	24	56.10 ± 7.5	51.4 ± 7.1	0.001
SBP (mmHg)	0	125.07 ± 4.2	126.6 ± 5.4	0.649
	24	124.8 ± 7.6	125.9 ± 7.4	0.126
DBP (mmHg)	0	84.1 ± 2.1	85.2 ± 7.0	0.138
	24	84.1 ± 4.5	84.7 ± 3.5	0.793

**Figure 2:**
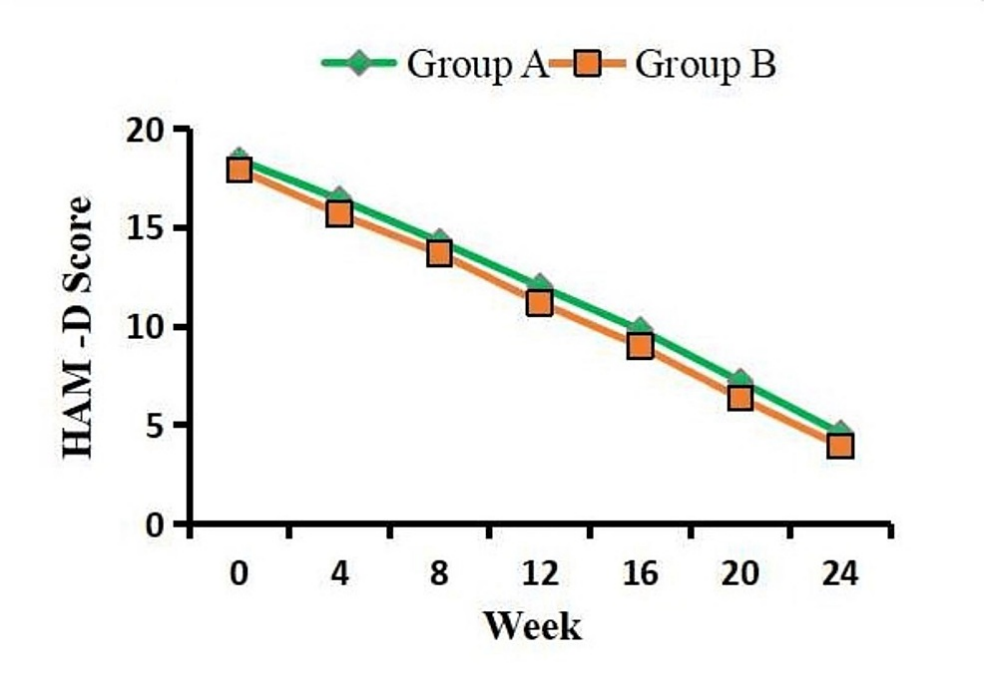
Comparison of clinical efficacy between the two groups using the HAM-D score. Values are presented as mean ± SD. Group A: vortioxetine 10 mg. Group B: fluoxetine 20 mg. HAM-D: Hamilton Depression Rating Scale

A statistically insignificant difference (p = 0.337) was noted in the overall remission of depression between the two groups over a 24-week treatment period. The remission rates observed in this study were 74.21% for vortioxetine (n = 60) and 75.50% for fluoxetine (n = 62) following a 24-week treatment period. Furthermore, on within-group analysis, both study drugs were effective in reducing the depression (HAM-D) score from baseline until week 24. Of note, fluoxetine showed a non-significant superiority in the reduction of the HAM-D mean score compared to vortioxetine (Figure 2).

**Figure 3:**
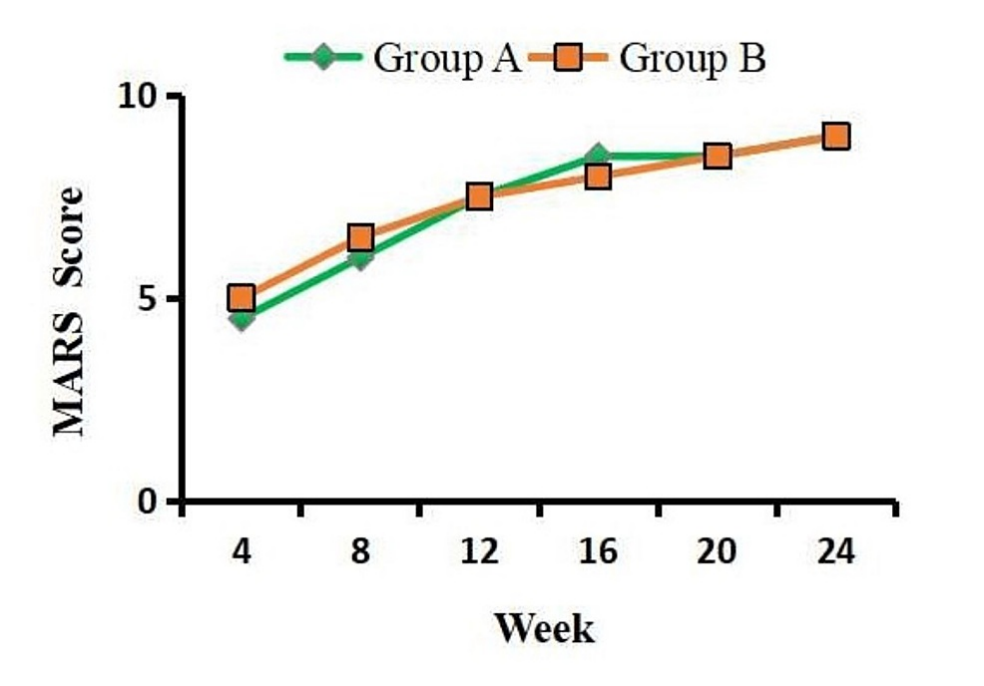
Comparison of medication adherence between the two groups using the MARS questionnaire. Values are presented as mean ± SD. Group A: vortioxetine 10 mg. Group B: fluoxetine 20 mg. MARS: Medication Adherence Rating Scale

The results of the independent-sample t-test analysis showed no significant difference in the MARS scores between the two groups at baseline (p = 0.121) and week 24 (p = 0.325) (Figure 3).

The incidence of ADRs was higher in group A (26.7%) compared to group B (17.7%). Group A reported five different ADRs, including diarrhea (2, 3.3%), weight loss (2, 3.3%), insomnia (4, 6.6%), gastric ulcers (6, 10%), and nausea (2, 3.3%). On the other hand, group B reported four different ADRs, namely, headache (3, 4.8%), dizziness (2, 3.2%), gastric irritation (3, 4.8%), and weight gain (2, 3.2%). The chi-square test did not find a statistically significant correlation between the two groups (p = 0.211). To assess the reported adverse events, a panel of three judges was formed. This panel consisted of psychiatrists, pharmacologists, and clinical pharmacists. The panel members conducted a causality assessment using the WHO causality scale and determined that most of the ADRs, such as diarrhea (3.3%), weight loss (1.6%), insomnia (3.3%), gastric ulcers (6.6%), nausea (1.6%), headache (4.8%), dizziness (1.61%), gastric irritation (3.2%), and weight gain (1.65%) were possibly related to the study medications, and the patients were treated for their ADRs. Of note, none of the patients withdrew their consent from the study due to experiencing ADRs. Furthermore, there was a statistically insignificant (p > 0.05) and only numerical improvement in SBP and DBP in both study groups.

## Discussion

MS is a cluster of risk factors that, if present together, increase the likelihood of developing cardiovascular and metabolic abnormalities such as dyslipidemia, hypertension, and hyperglycemia [14].

The clinical efficacy of vortioxetine and fluoxetine in mitigating depression has been demonstrated in various RCTs involving adult participants [1,14,19,20]. The existing body of literature fails to provide definitive findings regarding the impact of vortioxetine or fluoxetine on MS or its related symptoms. Previous reviews solely examined the effects of body weight and HbA1c improvement [21]. In contrast, our study delved into the impact of vortioxetine and fluoxetine treatment on various individual MS parameters, including waist circumference, body weight, FPG, TGs, HDL, and BP, in patients with MDD. Individuals who are diagnosed with MDD often demonstrate an increased sensitivity to stress, which can potentially result in more pronounced disturbances in glucose regulation. Our study found significant changes in fasting FPG levels; specifically, vortioxetine treatment led to decreased FPG levels, while fluoxetine treatment resulted in increased FPG levels [17,22]. Nevertheless, it ought to be noted that a few studies have presented conflicting results, indicating that fluoxetine could potentially have a positive impact on alleviating symptoms of depression and improving glycemic control [21,23].

Raeder et al. reported an association between dyslipidemia and the use of antidepressants [15]. Notably, both study drugs showed a significant increase in TG levels, consistent with prior research suggesting a link between SSRIs and elevated TG levels [23]. However, it is worth noting that several studies have reported no significant clinical changes in TGs associated with vortioxetine therapy [17,20,22]. Conversely, an interesting finding emerged from a study that demonstrated a significant decrease in TGs following fluoxetine treatment [21].

In line with previous findings, it was observed that fluoxetine administration led to a reduction in HDL levels [24]. In our study, we observed a noteworthy enhancement in the average score after 24 weeks of vortioxetine treatment. However, it is crucial to acknowledge that no specific research has been conducted thus far to establish a correlation between the effects of vortioxetine and HDL levels.

Numerous epidemiology investigations have documented that SSRIs appear to have no significant association with elevated SBP or DBP in individuals diagnosed with MDD [14,22,25-27]. The current investigation revealed a lack of statistically significant correlations between SBP and DBP following the administration of vortioxetine and fluoxetine. In contrast, fluoxetine may potentially exert a favorable impact on BP, particularly by reducing average BP levels, possibly through the inhibition of the autonomic nervous system [28]. Furthermore, Serodio et al. suggested that fluoxetine treatment does not pose any detrimental effects in association with SBP [29]. Indirect indicators of obesity are often employed for research and clinical use, and these measures employ various combinations of variables, such as body weight, age, and waist circumference [30]. This study revealed a noteworthy improvement in waist circumference, which stands in contrast to previous studies that reported no significant alterations in waist circumference following treatment with either vortioxetine or fluoxetine [17,19,20,24]. The efficacy of both vortioxetine and fluoxetine in alleviating depression, as measured by the HAM-D, was observed following a 24-week treatment period. The results of our study on fluoxetine have demonstrated a non-significant improvement in achieving remission of depression compared to vortioxetine. However, this finding contradicts the conclusions drawn by several other studies [16,17,22,25,27].

Vortioxetine and fluoxetine were well tolerated, with most ADRs being mild or moderate in intensity, and no dropouts were noted during the treatment period [22].

Our study had a few limitations. First, the study was designed as an open-label study. Second, we were unable to collect data from individuals under the age of 18 years, as they were deliberately excluded from the study population. Finally, it is important to note that the study was conducted at a single center, possibly exposed to the particular characteristics of the specific institution.

## Conclusions

In our study, patients receiving vortioxetine and fluoxetine had a significant association with FPG, HDL, TGs, and waist circumference. We conclude that vortioxetine is superior to fluoxetine in improving FPG, HDL, and waist circumference, whereas both drugs showed increased TG levels. Moreover, only numerical improvements were observed in SBP and DBP in both groups, but they were not significant. Regarding the remission rate of MDD, no significant difference was found between fluoxetine and vortioxetine. Overall, both study drugs were effective in reducing the HAM-D score, and both groups were comparable to each other in terms of medication adherence and ADRs.
